# Changes in physicochemical and anticancer properties modulated by chemically modified sugar moieties within sequence-related G-quadruplex structures

**DOI:** 10.1371/journal.pone.0273528

**Published:** 2022-08-23

**Authors:** Carolina Roxo, Anna Pasternak

**Affiliations:** Department of Nucleic Acids Bioengineering, Institute of Bioorganic Chemistry, Polish Academy of Sciences, Poznan, Poland; National Cancer Institute at Frederick, UNITED STATES

## Abstract

We systematically investigated the influence of locked nucleic acid (LNA), unlock nucleic acid (UNA), and 2’-O-methyl-RNA (2’-O-Me-RNA) residues on the thermal stability, structure folding topology, biological activity and enzymatic resistance of three sequence-related DNA G-quadruplexes. In order to better understand the mechanism of action of the studied modifications, a single-position substitution in the loops or G-tetrads was performed and their influence was analyzed for a total of twenty-seven modified G-quadruplex variants. The studies show that the influence of each modification on the physicochemical properties of G-quadruplexes is position-dependent, due to mutual interactions between G-tetrads, loops, and additional guanosine at 5’ or 3’ end. Nevertheless, the anticancer activity of the modified G-quadruplexes is determined by their structure, thus also by the local changes of chemical character of sugar moieties, what might influence the specific interactions with therapeutic targets. In general, UNA modifications are efficient modulators of the G-quadruplex thermodynamic stability, however they are poor tools to improve the anticancer properties. In contrast, LNA and 2’-O-Me-RNA modified G-quadruplexes demonstrated certain antiproliferative potential and might be used as molecular tools for designing novel G-quadruplex-based therapeutics.

## Introduction

G-quadruplexes (G4s) are non-canonical nucleic acid structures formed by guanine-rich oligonucleotides of RNA or DNA type [1–3]. They have the capacity to form G-tetrads through the assembly of four guanosine residues in a planar arrangement, stabilized by Hoogsteen hydrogen bonding. The stacking of two or more G-tetrads is one of the main characteristics of G-quadruplex structures. Importantly, DNA G-quadruplexes can exhibit outstanding variations in the molecular folding and topology, *i*.*e*. various molecularity, length and type of loops, guanosine base orientation, directionality of the strands, and the number of G-tetrads.

G-quadruplex forming sequences are present in the human genome and play important roles in pivotal biological processes within cells such as replication, transcription, and translation [4, 5]. They are also frequently found in the promoter regions of cancer‐related genes. In the last few years, many synthetic G-quadruplex forming sequences have been developed, demonstrating high affinity toward important proteins regulating gene expression and becoming a potent therapeutic tool [5, 6]. However, the prediction or design of a particular G-quadruplex structure with optimal characteristics for a desired therapeutic effect is still challenging [7–9]. One of the biggest challenges connected with G-quadruplex based drugs as well as with oligonucleotide-based therapeutics is their cellular instability. This limitation might be overcome by chemical modification of G-quadruplexes. Development in nucleic acids chemistry has been a key factor for the progress of modern molecular biology, biomedicine and biochemistry [10]. Moreover, the incorporation of modified nucleotides can improve not only G-quadruplexes lifetime but also their thermodynamic stability, cellular uptake and the affinity to target molecules [7, 11–13].

Among different chemical modifications of sugar moiety, the unlock nucleic acid (UNA), locked nucleic acid (LNA), and 2’-O-methyl-RNA (2’-O-Me-RNA) have attracted great attention and their influence on the G-quadruplex structure and thermodynamic stability has been extensively studied [11, 14, 15]. UNA as a 2’,3’-seco-RNA monomer is an acyclic RNA mimic which increases the flexibility of oligonucleotides leading to destabilization of duplexes [16]. However, incorporation of UNAs can increase thermodynamic stability of G-quadruplexes in a position dependent manner [14]. One of the most commonly modified G-quadruplex structure is thrombin binding aptamer (TBA). The substitution of thymidine by UNA-U in specific positions of the TBA loops stabilizes the G-quadruplex structure, whereas UNA-G placed within the G-tetrad destabilizes the structure or even totally prevents the G-quadruplex formation [14]. Agarwal et al. reported that UNAs have potential to improve the serum stability of G-quadruplex structures [17]. In contrast, LNA consists in a ribonucleotide analogue with a 2’-O-4’-C-methylene linkage and constitutes one of the strongest stabilizers of nucleic acids duplexes [18]. The introduction of single LNA into TBA G-quadruplex can destabilize or stabilize the structure in a position dependent manner and can change the biological activity by disrupting the TBA interactions with thrombin [19, 20]. Moreover, it was reported that LNA-G residues are generally tolerated within G-tetrads when they substitute positions that were originally occupied by *anti* conformers of guanosine [7, 21, 22]. Edwards et al. reported that the insertion of three LNA monomers at specific sites of the G-quadruplex forming V7t1 aptamer can improve is stability and antiproliferative activity in human breast cancer cells [23]. Similarly, like UNA and LNA, also the 2’-O-Me-RNA substituted in place of guanine nucleotides can increase the stability or disrupt the G-quadruplex structure, being dependent on the glycosidic conformation of the replaced guanosine [24, 25]. Single substitution of the *syn*-oriented guanosine within TBA G-tetrad by 2’-O-Me-RNA-G caused structure destabilization, whereas the presence of this modification at *anti*-position preserved G-quadruplex structure in K^+^ environment [25].

**Fig 1:**
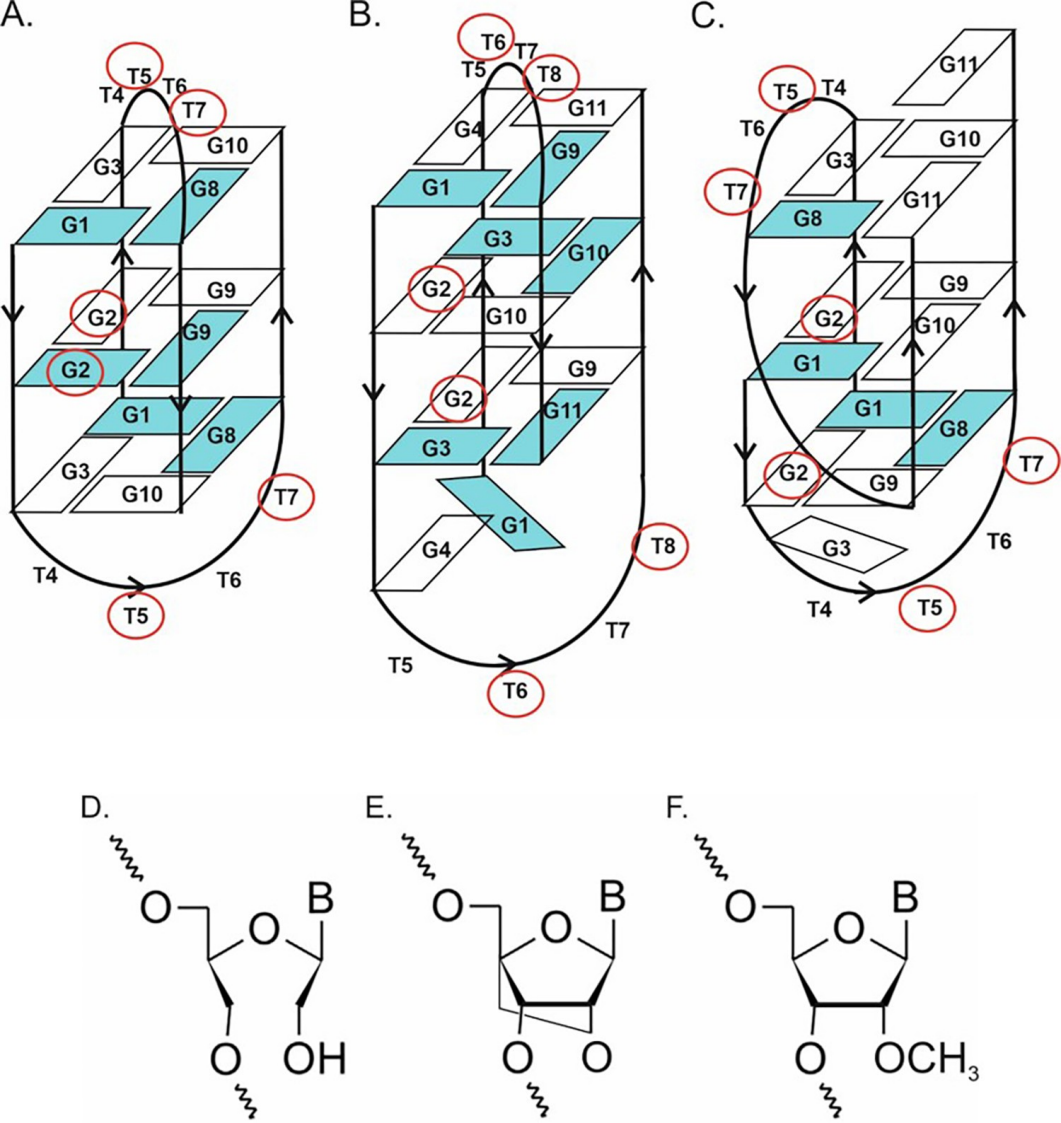
The DNA G-quadruplex structures formed by: **ON1** (A), **ON2** (B), **ON3** (C) and the chemical modifications applied in the studies: Unlock Nucleic Acid (UNA) residue (D), Locked Nucleic Acid (LNA) residue (E), 2’-O-methyl-RNA (2’-O-Me-RNA) residue (F). Blue color within G-quadruplex structures represents *syn-*conformation of guanosines whereas red circles indicate the positions of chemical modifications. According to Črnugelj, M. et al. [29, 30].

Herein we present a systematic study on the influence of UNA, LNA, and 2’-O-Me-RNA on thermodynamic stability, biological activity and enzymatic resistance of three sequence-related DNA G-quadruplexes which show considerable antiproliferative properties against HeLa cancer cells [8]. The structures formed by oligonucleotides consist of three G-tetrads and loops containing four thymidine residues. Moreover, two of the variants vary in the presence of additional guanosine at 5’ or 3’ end. The majority of the studies published so far on the influence of sugar-based modifications were performed for TBA as a model G-quadruplex structure [11, 19, 20, 26]. The studies based on one type of G-quadruplexes barely demonstrate the general influence of the chemical modifications when applied to other G-quadruplex structures. Therefore, it is important to verify the influence of these modifications on different types of G-quadruplexes. The comprehensive investigations of the influence of the above chemical modifications within sequence related structures assures better understanding of their mechanism of action. In order to study how UNA, LNA, and 2’-O-Me-RNA residues influence the G-quadruplex structures and their biological properties, a single substitution was made in specific positions of the loops and G-tetrads (Fig 1, Table 1). The systematic studies presented in this article disclose important considerations and understandings for the use of the sugar-based chemical modifications in the G-quadruplex structures and might facilitate the design and development of specifically modified G-quadruplexes for therapeutic applications.

**Table 1:** Thermal stability of G‐quadruplexes^a^.

Name	Sequence (5’– 3’)	T_M_^b^ (°C)	ΔT_M_(°C)
**ON1**	GGGTTTTGGG	45.0	**0**
**ON2**	GGGGTTTTGGG	53.0	**0**
**ON3**	GGGTTTTGGGG	56.6	**0**
**LNA Modification**			
**ON1 –L2**	G G^L^ GTTTTGGG	58.0	+13.0
**ON1 –L5**	GGGTT^L^TTGGG	42.4	-2.6
**ON1 –L7**	GGGTTTT^L^GGG	46.7	+1.7
**ON2 –L2**	G G^L^ GGTTTTGGG	59.1	+3.1
**ON2 –L6**	GGGGTT^L^TTGGG	49.3	-3.7
**ON2 –L8**	GGGGTTT T ^L^ GGG	54.2	+1.2
**ON3 –L2**	G G^L^ GTTTTGGGG	60.5	+3.9
**ON3 –L5**	GGGT T^L^TTGGGG	60.0	+3.4
**ON3 –L7**	GGGTTT T^L^GGGG	55.2	-1.4
**UNA Modification**			
**ON1 –U2**	GG^U^GTTTTGGG	15.6	-29.4
**ON1 –U5**	GGGTU^U^TTGGG	46.1	+1.1
**ON1 –U7**	GGGTTTU^U^GGG	43.1	-1.9
**ON2 –U2**	GG^U^GGTTTTGGG	35.8	-13.5
**ON2 –U6**	GGGGTU^U^TTGGG	54.0	+1.0
**ON2 –U8**	GGGGTTTU^U^GGG	52.3	-0.8
**ON3 –U2**	GG^U^GTTTTGGGG	25.8	-30.8
**ON3 –U5**	GGGTU^U^TTGGGG	60.3	+3.7
**ON3 –U7**	GGGTTTU^U^GGGG	51.3	-5.3
**2’-O-Me-RNA Modification**			
**ON1 –M2**	GG^M^GTTTTGGG	44.9	-0.1
**ON1 –M5**	GGGT U^M^ TTGGG	45.9	+0.9
**ON1 –M7**	GGGTTT U^M^ GGG	43.9	-1.1
**ON2 –M2**	GG^M^GGTTTTGGG	58.6	+5.6
**ON2 –M6**	GGGGT U^M^ TTGGG	50.6	-2.4
**ON2 –M8**	GGGGTTT U^M^ GGG	51.1	-1.9
**ON3 –M2**	GG^M^GTTTTGGGG	54.8	-1.8
**ON3 –M5**	GGGT U^M^ TTGGGG	56.9	+0.3
**ON3 –M7**	GGGTTT U^M^ GGGG	55.9	-0.7

^a^—100 mM KCl, 20 mM sodium cacodylate, 0.5 mM EDTA(Na)_2_ (pH 7.0)

^b^—calculated for 10^−4^ M concentration. Full thermodynamic data is presented in, S2 Table in S1 File.

## Materials and methods

### Chemical synthesis of oligonucleotides

The synthesis of the oligonucleotides listed in Table 1 were performed on an automatic RNA/DNA synthesizer using the standard phosphoramidite approach with commercially available phosphoramidite building blocks. The deprotection steps were performed according to previously used and described protocols [27, 28]. The composition of all oligonucleotides was confirmed by MALDI‐TOF (Bruker Autoflex, Billerica, MA, USA) mass spectrometry (S1 Table in S1 File).

### UV melting studies

UV melting analysis was accomplished for nine different concentrations of each oligonucleotide in the range of 10^−4^ to 10^−6^ M. A specific amount of each oligonucleotide was evaporated to dryness and dissolved in buffer containing 100 mM potassium chloride (KCl), 20 mM sodium cacodylate and 0.5 mM Na_2_EDTA (pH 7.0). The concentrations of single‐stranded oligonucleotides were firstly calculated based on the stock solutions absorbance at 85°C. The extinction coefficients were calculated using the OligoAnalyzer tool (Integrated DNA Technologies). Absorbance versus temperature curves were acquired using the UV melting method at 295 nm with the temperature range of 95°C to 3°C and a temperature decrease of 0.2°C/min using a JASCO V‐650 (Cremella (LC) Italy) spectrophotometer equipped with a thermoprogrammer. The thermodynamic parameters were analyzed and determined using MeltWin 3.5 software. The melting temperatures calculated for the 10^−4^ M concentration of the oligonucleotide are denoted as T_M_.

### Circular dichroism spectra

The CD spectra were collected using the JASCO J‐815 (Cremella (LC) Italy) spectropolarimeter. Each oligonucleotide was evaporated to dryness and dissolved in 1ml buffer containing 100 mM KCl, 20 mM sodium cacodylate and 0.5 mM Na_2_EDTA (pH 7.0) to reach a sample concentration of 3.0 μM. The G‐quadruplex samples were denatured at 90°C for 3 min and then cooled to room temperature overnight, followed by data collection. The spectra were recorded in triplicate in the 210–300 nm wavelength range, at 37°C and the buffer spectrum was subtracted from the sample spectra. Data analysis was made in the Origin v8.5 software.

### Cell culture

The human cervical adenocarcinoma (*HeLa*) cell line (ATCC, Rockville, MD, USA) was cultured in RPMI 1640 medium supplemented with 10% fetal bovine serum (FBS) (Gibco, Waltham, MA, USA), 1% Antibiotic–Antimycotic solution (Gibco, Waltham, MA, USA) and 1% MEM vitamin solution (Gibco, Waltham, MA, USA). The cells were grown in an incubator at 37°C with 5% CO_2_ and with humidity of 95%.

### Antiproliferative assay

The G‐quadruplexes antiproliferative properties were measured by using the MTT assay. Each oligonucleotide had a final concentration of 10 μM and was dissolved in 1× PBS buffer with 100 mM potassium chloride (KCl), followed by denaturation at 90°C for 5 min with subsequent cooling to room temperature overnight. The experiments were conducted on cervical HeLa cell line, being seeded in 96‐well plates at a density of 500 cells/well in 100 μl of RPMI 1640 medium (Gibco, Waltham, MA, USA) supplemented with 10% FBS (Gibco, Waltham, MA, USA) and MEM 1% vitamin solution (Gibco, Waltham, MA, USA). The 96‐well plates were incubated at 37°C, with 5% CO_2_ and a humidity of 95% for 24h. Next, HeLa cells were exposed to a 10 μM concentration of G‐quadruplex oligonucleotides for 7 days. Then, the growth medium was removed and 100 μl/well of 1x MTT solution (Sigma‐Aldrich, Darmstadt, Germany) in RPMI 1640 without phenol red media, was added to the wells. The cells were incubated at 37°C with 5% CO_2_ and 95% humidity for 4h. Following, the medium was removed and replaced with 100 μl/well solution of 70% isopropanol and 40 mM HCl to liquefy the blue‐purple crystals of formazan. The plates were shaken at 300 rpm at room temperature for 30 min. For the quantification of the free formazan a microplate reader xMark (Bio‐Rad, CA, USA) was used and the absorbance was measured at 570 nm. Data analysis was made using Microsoft Excel 2016 software. Cell viability was calculated according to the equation: % Viability = (mean OD of the cells with oligo/ mean OD of the control cells) x 100. Each experiment was repeated in triplicate, and the results were expressed as the means ± SD.

### Nuclease stability assay

In order to analyze the stability tendency of the G-quadruplex, a nuclease stability assay was conducted by CD spectroscopy method. Approximately 7 nmol of stock solution of each G-quadruplex was evaporated to dryness, dissolved in 1xPBS with 100mM potassium chloride (KCl) buffer, denatured 5min at 90°C and then cooled to room temperature overnight. The degradation patterns were analyzed by monitoring the CD signal decrease of each sample in 400 μL of RPMI 1640 medium (Gibco, Waltham, MA, USA) with 10% fetal bovine serum (FBS) (Gibco, Waltham, MA, USA) and incubated at 37°C. The CD spectra were recorded at 4°C using JASCO J‐815 (Cremella (LC) Italy) spectropolarimeter equipped with a Peltier temperature control system after 0h and 24h of incubation. The CD spectra were recorded in duplicate in the 235–330 nm wavelength range. Each spectrum presented was corrected for the spectrum of the reaction medium (RPMI with 10%FBS). Data analysis was performed using Origin v8.5 software where the maximum CD signal was obtained for the 0 and 24h. The stability percentage of the G-quadruplexes was calculated in Excel, where the signal intensity at 0 time corresponded to 100% of oligonucleotide viability.

### Statistical analysis

The results are reported as the means ± standard deviation, and at least 3 independent biological replicates were performed for the MTT assay. Data analysis was made using Sigma Plot software (version 12.5; SysTest Software Inc., El Segundo, CA, USA), and the statistical significance treatment in cells between the native sequence and modified sequences was tested by one‐way ANOVA. Normality was tested by the Shapiro–Wilk test. The differences were considered statistically significant for p < 0.001.

## Results and discussion

Diverse nucleic acid chemistries, as well as various G-quadruplex architectures, make designing of an optimal G-quadruplex therapeutic agent relatively challenging. In this article, we selected three G-quadruplex forming sequences (Fig 1) from a pool of five, previously studied oligonucleotides [8]. The G‐quadruplexes were selected based on the similarities of the main G-quadruplex forming structural elements, *i*.*e*. loops composition and number of G-tetrads, and according to the considerable inhibitory effect on the HeLa cancer cells growth. Moreover, all of the oligonucleotides showed moderate stability in human serum, making them good candidates for improving their therapeutic potential, most probably due to the synergistic toxicity of G-quadruplex degradation products [8]. For further studies we selected three commonly used chemical modifications of sugar residues *i*.*e*. UNA, LNA and 2’-O-Me-RNA which were reported previously as modulators of the TBA G-quadruplex physicochemical and biological properties. Importantly, the studies based on isosequential G-rich oligonucleotides with minor structural variations give an unprecedented opportunity to understand more globally the influence of chemical modifications on G-quadruplex structure and biological activity.

According to previously published NMR studies, oligonucleotide d(G_3_T_4_G_3_)_2_ (**ON1**) forms an antiparallel, dimeric G‐quadruplex with three G‐tetrads and diagonal loops (Fig 1A) [29, 30]. The G-quadruplex core exhibits *syn*-*syn*-*anti*-*anti* alternations inside G-quartets. The folding topology of d(G_4_T_4_G_3_)_2_ (**ON2**, Fig 1B) in Na^+^ solution consists of a bimolecular, antiparallel, diagonally looped G‐quadruplex with three G‐tetrads presenting similar to **ON1** *syn-syn-anti-anti* alternation within each G-quartet. This G‐quadruplex structure is asymmetric due to two guanine residues aligned on one side of the G‐quadruplex core [29, 30]. The d(G_3_T_4_G_4_)_2_ (**ON3**, Fig 1C) folds into a bimolecular, asymmetric G-quadruplex with two G‐tetrads with *syn-anti-anti-anti* and one G-tetrad with *syn-syn-anti-anti* alternation inside G-quartets. This G-quadruplex has two different types of loops *i*.*e*. diagonal and edge types. Moreover, two guanosine residues (G3 and G11) from one of the strands are positioned outside the G-quadruplex core [29].

G-quadruplex formation of modified oligonucleotides was evaluated by UV melting analysis, providing comprehensive thermodynamic parameters of the twenty-seven modified variants and information about stoichiometry of the folding process. In order to study the effect of the chemical modifications on the G-quadruplex topology, the circular dichroism (CD) spectroscopy was also performed. The biological studies were performed by MTT assay in HeLa cancer cell line to investigate the antiproliferative activity of the modified variants. The nuclease stability was monitored by CD measurements to evaluate oligonucleotides viability tendency after 24h incubation in RPMI medium supplemented with 10% FBS. The extensive variety of analyses permitted us to analyze the influence of the UNA, LNA and 2’-O-methyl-RNA modifications on the G-quadruplex structure, topology, anticancer potential, and enzymatic viability.

### The influence of modified residues on thermodynamic stability of G-quadruplexes

UV melting experiments were performed to verify the influence of a single substitution of the nucleoside residues on the thermodynamic stability of the main G-quadruplex sequences. The sugar-based modifications *i*.*e*. LNA, UNA and 2’-O-Me-RNA were placed in three different positions of **ON1**, **ON2** and **ON3** G-quadruplexes (Fig 1A–1C). The molecularity folding of the studied G‐quadruplex structures was analyzed through the comprehensive analysis of the Tm vs. sample concentration dependence.

The selected G-quadruplexes differ slightly in structure, having in common three G-tetrads in the core and loops containing four thymidine residues. Previously it was proven that **ON1** is less thermodynamically stable (T_M_ value was 44.6°C) in comparison with **ON2** and **ON3** (T_M_ 53.0 and 56.6°C, respectively) due to the lack of additional guanosine residues at the terminal positions (Fig 1A) [8]. Oligonucleotide **ON2** is a variant of **ON1** with extra guanosine at the 5’ end and the only G-quadruplex in this set whose folding is reported as being cation-dependent [30]. In the presence of Na^+^ the G1 residue from one strand and G4 residue from the other strand are not directly involved in G‐tetrad formation, being aligned at the same side of the G-quadruplex core spanned by two diagonal loops (Fig 1B) [30]. In the K^+^ solution **ON2** is suggested to be more polymorphic, however it might be assumed that the specific structure observed in Na^+^ is predominant or at least present as one of the possibilities. **ON3** is also a variant of **ON1** with extra guanosine at the 3’ end, forming a different bimolecular G-quadruplex topology (Fig 1C). In one of the **ON3** strands, G3 and G11 guanosines are positioned at opposite sides of the G‐quadruplex core, whereas all guanosine residues in the second **ON3** strand are involved in G‐tetrad formation [29]. Melting analysis of **ON1**-**ON3** and their modified variants revealed a dependence between T_M_ values versus sample concentrations for all oligonucleotides indicating that the studied G-quadruplex structures are folded intermolecularly.

**Fig 2:**
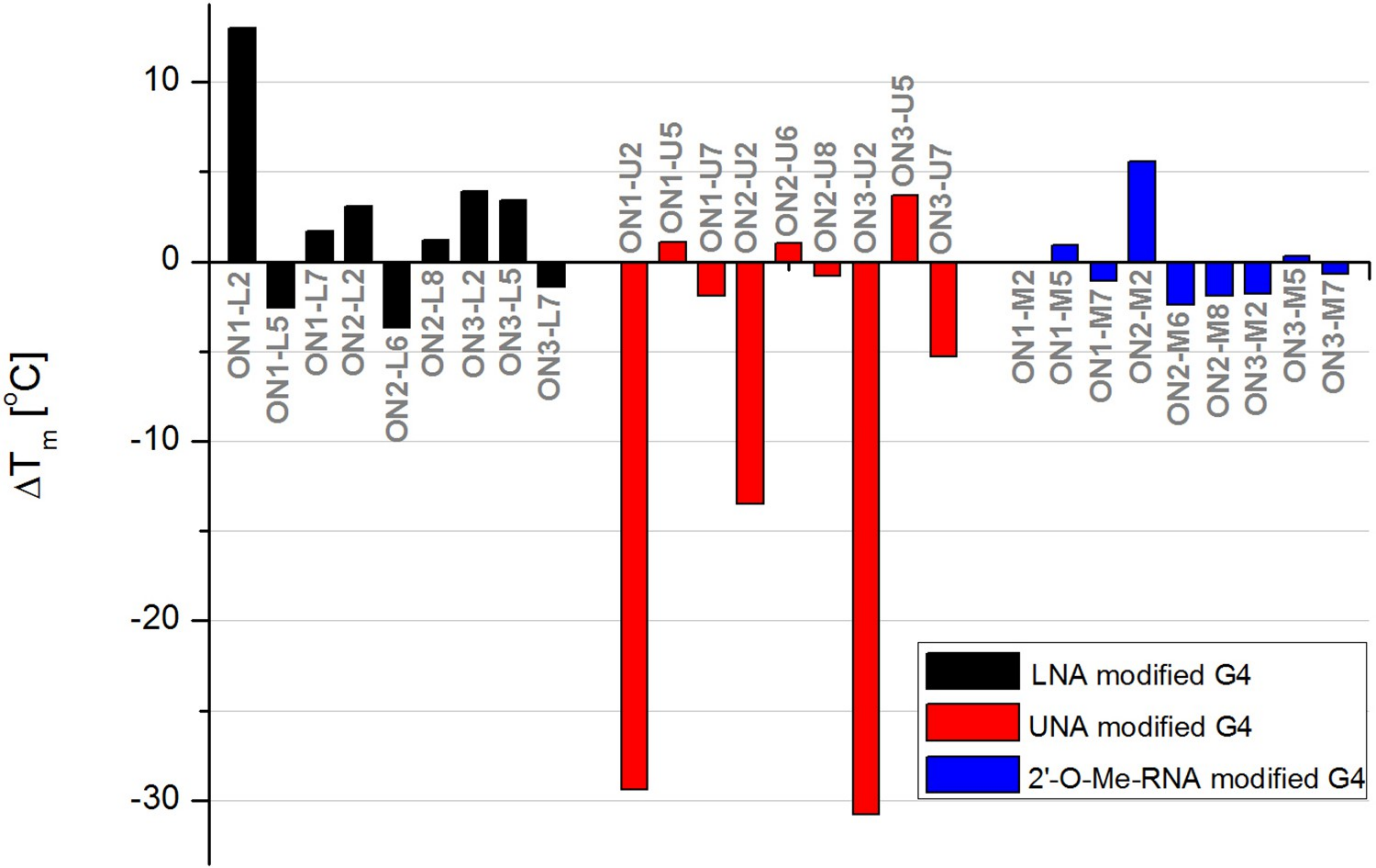
The influence of chemical modifications on thermal stability of DNA G-quadruplex structures formed by: ON1, ON2, and ON3. Positive ΔTm values correspond to stabilization, negative values reflects destabilization of G-quadruplex structure.

The replacement of guanosine with LNA-G in the G-tetrad (G2 position) caused stabilization of the three G-quadruplex variants leading to an increase in T_M_ values by 13.0°C, 3.1°C, and 3.9°C for **ON1–L2**, **ON2–L2**, and **ON3–L2**, respectively (Table 1, Fig 2). It was reported that tolerance of G-tetrads towards LNA modifications is dependent on the *syn*/*anti* glycosidic bond conformation of the replaced guanosine residues [7, 21, 22]. When the LNA–G replaces guanosine in *anti-*conformation the increase in thermal stability was observed. Interestingly, in our studies the G2 position substituted by LNA-G within **ON1** was originally occupied by *anti* and *syn* guanosine conformers, whereas in **ON3** and presumably also in **ON2** only *anti* position was modified. Therefore, the large difference in thermal stability between **ON1–L2** vs. **ON2–L2** and **ON3–L3** cannot be rather due to the type of glycosidic conformation of the substituted guanosines, as previously reported, referring to the favorable effect of LNA modification on *anti-*position only [7, 20, 24]. The LNA-G modifications in **ON1–L2** G-quadruplex occupy adjacent positions of the internal G-tetrad, whereas in **ON2–L2** and **ON3–L2** the diagonal localization of LNA–Gs within two different G-tetrads is rather observed, what might be responsible for the various thermal effects induced by LNAs within studied structures (Fig 1A–1C). Different effects can be also a result of G-quadruplex topology changes, as described in the next section. The replacement of thymidine inside the loop of **ON1–L5** (position T5) and **ON2–L6** (position T6) is slightly disadvantageous for the G-quadruplex thermal stability leading to decrease of T_M_ value by 2.6°C and 3.7°C, respectively. However, the presence of LNA-T at position T5 of **ON3–L5** increases G-quadruplex thermal stability (ΔT_M_ = +3.4°C). The replacement of thymidine by LNA-T in the loop near the guanosine residue (position T7 of **ON1**, position T8 of **ON2**) was favorable for the G-quadruplex thermal stability increasing T_M_ value by 1.7°C for **ON1–L7** and 1.2°C for **ON2–L8**. In contrast, a decrease in melting temperature by 1.4°C was caused by the presence of LNA-T at position T7 of **ON3–L7**. The unmodified **ON1–ON3** G-quadruplex structures are characterized by a number of interactions between the thymidine residues in the loops *i*.*e*. stacking interactions and H-bonding between each other or with G-quartet residues [29–31]. The different effect of LNA–T at position T5 of **ON3** in reference to **ON1** and **ON2** might be due to more favorable orientation of LNA–T for the interactions within **ON3** or the fact that T5 from both strands are not involved in the additional interactions with G-tetrad resulting *e*.*g*. in less significant disruption of the structure. In contrast, in **ON1** and presumably also in **ON2** the thymidine residues at T5 and T6 positions of both strands are involved in various stacking interactions and H-bonding, whereas the effect is opposite for the T7 and T8 positions. In this case, the thymidine residues of all studied G-quadruplexes in both strands are involved in the variety of interactions with the remaining residues within the structure. Therefore, the final effect of the LNA–T substitution might be dependent on the character of interactions and on the favorable or unfavorable orientation of the LNA–T residue forced by locked nature of the modification or the topology change induced by LNAs (see next section).

In general, the presence of LNA-T modification within a loop, results more often in a slight enhancement of the thermal stability of G-quadruplex structures when the modification is positioned near the G-tetrad. However, the most significant improvement of the G-quadruplex thermal stability occurred when the LNA was placed directly within G-tetrad.

As expected, the replacement of guanosine residue in the G-tetrad by UNA-G at G2 position was highly disadvantageous for thermal stability of **ON1**, **ON2** and **ON3** G-quadruplexes (Table 1, Fig 2). We observed similar destabilization for **ON1–U2** and **ON3–U2** (ΔT_M_ = -29.4°C and -30.8°C, respectively), whereas the decrease of **ON2–U2** melting point in reference to **ON2** was -13.5°C. This could be due to the presence of specific G-quadruplex structure of **ON2–U2**, where two guanosine residues are aligned at the same side of the G-tetrad, mimicking almost half of the G—tetrad and providing additional stabilizing element which neutralizes unfavorable consequences of the presence of UNAs. However, the influence of folding topology changes revealed by CD spectra analysis described below should be also considered. Agarwal et al. reported that substitution of single UNA modification in the G-tetrad results in considerable destabilization of the structure with significant decrease in melting temperature [17]. Thus, the negative impact of UNA-G within G-tetrad is probably due to distortion of stacking interactions and in consequence to the destabilization of the entire structure. Interestingly, the presence of UNA-U inside the loops (positions T5 of **ON1** and **ON3** or T6 of **ON2**) demonstrated to be thermally advantageous for all studied G-quadruplexes (ΔT_M_ = +1.1°C for **ON1–U5**, +1.0°C for **ON2-U6** and +3.7°C for **ON3–U5**). In contrast, the substitution of thymidine residue at position T7 (**ON1** and **ON3**) or T8 (**ON2**) resulted in decrease of thermal stability of **ON1** and **ON3** by 1.9°C (**ON1–U7**) and 5.3°C (**ON3–U7**), whereas destabilization of **ON2** was less significant (ΔT_M_ = -0.8°C, **ON2–U8**). Previously published data indicated that UNA modification in the loop stabilizes G-quadruplex structure and destabilizes when present in the core [14, 17]. Our studies suggest that only substitution of internal thymidine residues at positions T5 or T6 by UNA–U enhances the G-quadruplex thermal stability, whereas the presence of UNA residues at positions T7 or T8 leads to structure destabilization, what indicates that the thermal effect of UNAs within the G-quadruplex loops might be also position-dependent. Notably, the overall effects of the presence of LNAs and UNAs within **ON1–ON3** G-quadruplex structural elements are opposing *i*.*e*. UNA stabilizes the structure at the positions at which LNA is unfavorable and *vice versa*. This clearly suggests that flexibility or stiffness of sugar moiety is one of the pivotal factors for G-quadruplex structure stability.

Replacement of one of the guanosine residues in the G-tetrad by 2’-O-Me-RNA-G at G2 position caused a minor decrease in the thermal stability of **ON1** and **ON3** (Table 1, Fig 2, ΔT_M_ = -0.1°C for **ON1–M2** and -1.8°C for **ON3–M2**). In contrast, the presence of 2’-O-Me-RNA-G at G2 position of **ON2** increased the thermal stability by 5.6°C. This diverse behavior might be due to the differences in G-quadruplex structure of **ON1–ON3**, what stays in accordance with previous investigations indicating that 2′-O-methyl-RNA is able to variously modulate G-quadruplex stability depending on the type of the G-quadruplex folding [32]. However, the shift of G-quadruplex folding topology of **ON2–M2** can be also the contributor to various effects observed for the variants modified within the G-quadruplex core.

The thymidine replacement within the loops by 2’-O-Me-RNA residues at position T5 of **ON1** (**ON1–M5**) and **ON3** (**ON3–M5**) demonstrated only a slight increase in thermal stability of **ON1** and **ON3** (ΔT_M_ = +0.9°C for **ON1–M5**, +0.3°C for **ON3–M5**). In contrast, the presence of 2’-O-Me-RNA at position T6 of **ON2** (**ON2–M6**) caused a decrease in the thermal stability (ΔT_M_ = -2.4°C). Replacement of thymidine by 2’-O-Me-RNA in the loop near the guanosine residue at position T7 of **ON1** and **ON3** and at position T8 of **ON2** resulted in decrease of the G-quadruplex thermal stability by 1.1°C (**ON1–M7**), 1.9°C (**ON2–M8**) and 0.7°C (**ON3–M7**).

In general, the 2’-O-Me-RNA modification within G-tetrads induced mild changes in the thermal stability of studied G-quadruplexes, in comparison to LNA and UNA modifications. According to already published data, LNA and 2’-O-Me-RNA modifications adopt *C3’endo* sugar puckering, and both were reported to increase thermal stability of G-quadruplexes when they replace guanosine residue having *anti* conformation [24, 25]. The different outcome between these two chemical modifications in the G-tetrad might be due to the permanent lock of LNA sugar conformation, making it more rigid, while the 2’-O-Me-RNA residues are more flexible and can maintain the base stacking almost untouched [33].

Overall, the **ON2** G-quadruplex demonstrated to be more tolerant for chemical modifications in the G-tetrad, showing the most restrained thermal stability changes among all three types of studied G-quadruplexes. This might be due to the extra guanosine residue at the 5’ terminal position of oligonucleotide, which results in formation of G-quadruplex core with two guanosine residues aligned at the same side of the G-tetrad mimicking almost half of a G-tetrad and providing more stability to the G-quadruplex structure. Even though **ON3** includes the extra guanosine at the 3’ end, the positioning of two guanosine residues within G-quadruplex structure is at opposite sides of the core, not leading to extra stability as it is observed in **ON2**.

### Folding topology of modified G-quadruplexes

Circular dichroism spectroscopy is a primary technique for the characterization of G-quadruplex topology and is based on changed absorbance of circularly polarized light, providing different CD spectral characteristics [11, 34]. Various G-quadruplex topologies display unique CD spectra signatures and can be divided into three types. A parallel G‐quadruplex presents a CD spectrum with a positive band at 260−265 nm and a negative band at 240−245 nm. A G-quadruplex with antiparallel topology typically shows a positive peak at 290−295 nm and a weaker negative band at 260−265 nm, whereas hybrid G‐quadruplex topology is characterized by positive bands at 295 and 270 nm and a negative band at 240 nm. However, some exceptions to the above rules have been noted [35].

The CD spectra of d(G_3_T_4_G_3_)_2_ (**ON1**) and d(G_4_T_4_G_3_)_2_ (**ON2**) show reshaping of CD pattern which is typical for an antiparallel G-quadruplex topology with changed glycoside bond angle arrangement of guanosines in the G-tetrads or for structural polymorphism with a predominance of antiparallel folding topology. Our findings stay in accordance to previous reports [8, 35]. In contrast, the CD spectra of d(G_3_T_4_G_4_)_2_ (**ON3**) demonstrated a hybrid topology. The CD spectra of **ON1**, **ON2** and **ON3** variants containing LNA, UNA or 2’-O-Me-RNA residues were analyzed to determine the influence of a particular modification on the G-quadruplex topology.

**Fig 3:**
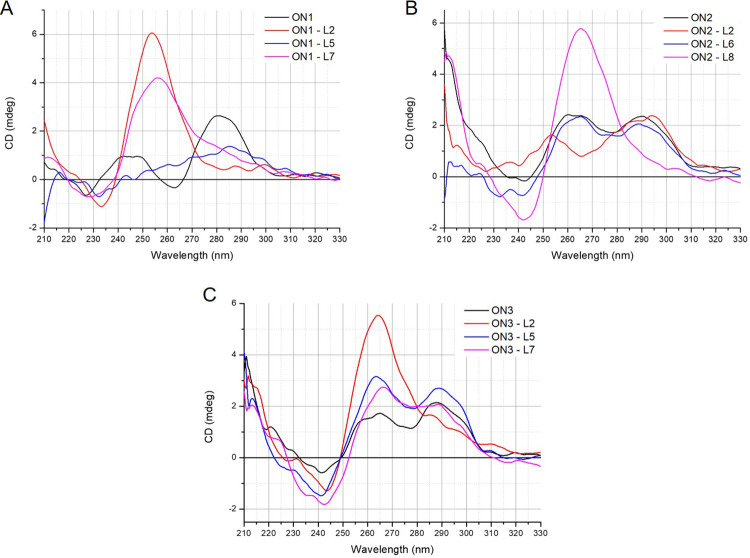
CD spectra of LNA-modified G-quadruplex variants.

**ON1** variants **ON1–L2** and **ON1–L7** demonstrated a shift of CD bands from the one which is characteristic for antiparallel topology towards a large positive band around 255 nm, resembling a parallel topology (Fig 3A). Surprisingly, a lack of any band characteristic for particular topologies was observed for **ON1–L5**, suggesting an absence of the structure even though thermal analysis indicated the presence of a relatively stable G-quadruplex (T_M_ = 42.4°C). The **ON2–L2** demonstrated a shift of the positive signal at 260 nm to around 250 nm, maintaining the positive signal at 295 nm (Fig 3B). The variant **ON2–L6** maintained the initial CD pattern of **ON2**, whereas **ON2–L8** demonstrated a shift of topology for parallel. The CD spectra of **ON3** variants modified with LNA (Fig 3C) revealed a shift of topology from hybrid to parallel only when LNA was placed at position G2 of G-tetrad (**ON3–L2**), though the LNA modifications in the loops did not change CD pattern of **ON3**. Previously reported studies indicate that LNA single substitution within G-tetrad leads to a change of G-quadruplex topology from antiparallel to parallel, being partially in accordance with our findings [22, 24].

**Fig 4:**
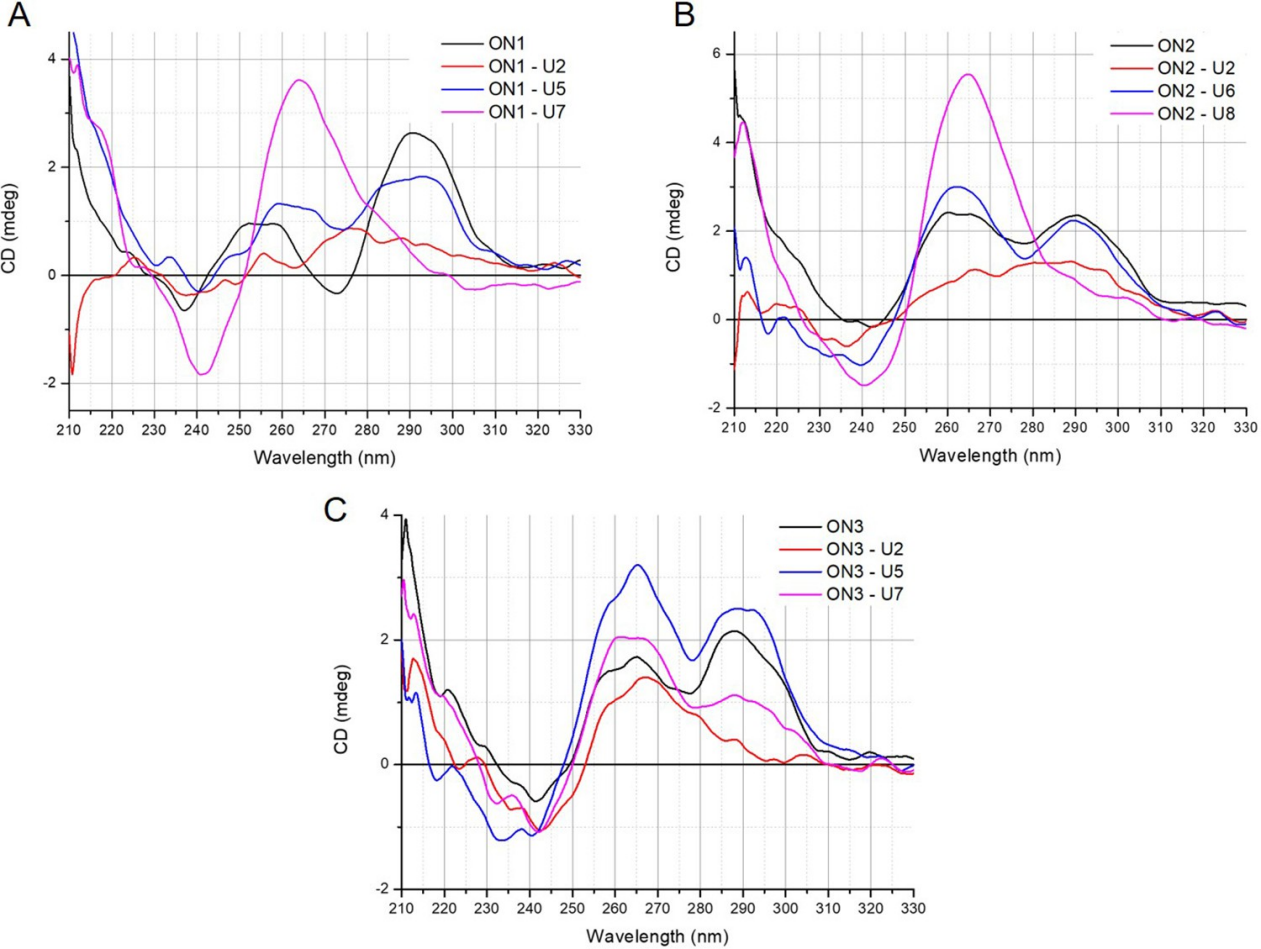
CD spectra of UNA-modified G-quadruplexes.

The presence of UNA modifications within G-tetrad of **ON1** (Fig 4A) caused the loss of characteristic CD bands. This could be expected due to low melting temperature of this G-quadruplex (**ON1– U2**, T_M_ = 15.6°C) being unfolded at the physiological temperature of 37°C. The CD spectra of **ON1–U5** clearly showed a shift of CD pattern presenting two maxima near 260 nm and 290 nm and a minor negative band near 240 nm. The above change might indicate a hybrid topology of **ON1–U5** G-quadruplex. In contrast, **ON1–U7** demonstrated a shift of topology from antiparallel to parallel. A significant reduction of CD bands was also observed for **ON2–U2** containing UNA residue within G-tetrad and showing low stability of G-quadruplex structure (Fig 4B). On the contrary, the substitution of thymidine residues within loops of **ON2** demonstrated no CD pattern change for **ON2–U6**, however for **ON2–U8** a shift from towards parallel topology was observed. Unexpectedly, the presence of UNA within G-tetrad of **ON3**, despite significant destabilization of the structure (ΔT_M_ = -30.8°C) induced only a shift of G-quadruplex topology from hybrid to parallel, with no significant signal reduction (Fig 4C). The remaining UNA-modified variants of **ON3** maintained the same topology as **ON3**.

**Fig 5:**
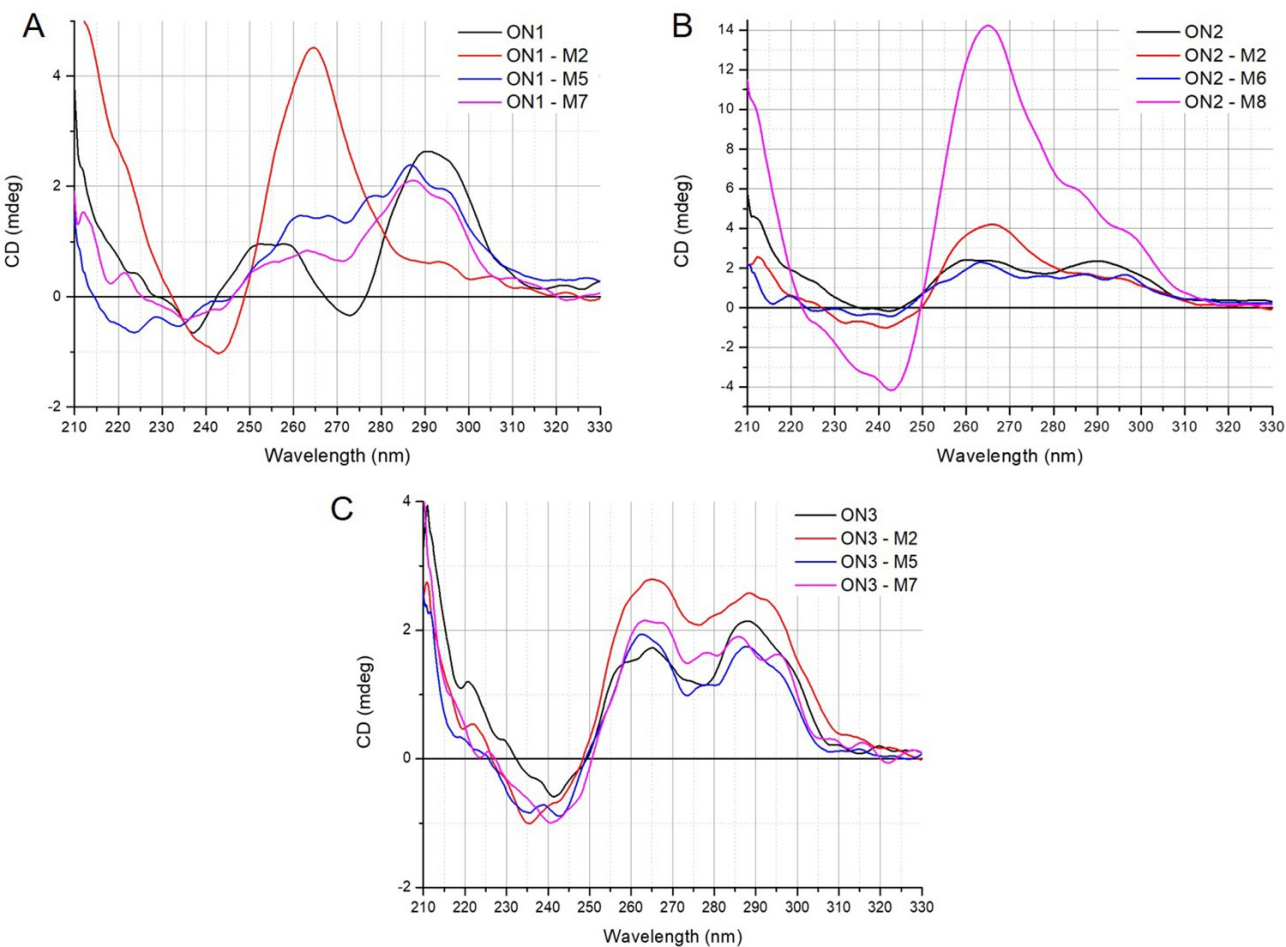
CD spectra of the 2’-O-Me-RNA-modified G-quadruplexes.

The presence of 2’-O-Me-RNA modification within G-tetrad of **ON1** induced a shift of topology from antiparallel to parallel (Fig 5A, **ON1–M2**). The CD spectra of **ON1–M5** and **ON1–M7** are also characterized by a minor shift of CD bands in reference to **ON1**, with two positive signals around 265 nm and 285 nm, however their topology is unclear. The CD spectra of **ON2** variants, *i*.*e*. **ON2–M2** and **ON2–M8** (Fig 5B) are characterized by main positive peak at 260 nm and minor positive signal at 295 nm, what indicates a parallel topology of G-quadruplex structure. The **ON2–M6** variant maintains a similar CD spectrum to **ON2**. Interestingly, CD patterns of **ON3** variants modified with 2’-O-Me-RNA (Fig 5C), did not demonstrate any topology changes.

The CD spectra analysis revealed that **ON1** and **ON2** G-quadruplexes are more predisposed to modification-induced topology changes. Remarkably, the original G-quadruplex structures of the **ON1** and to some extent also **ON2** are characterized by antiparallel topology, whereas **ON3** forms a hybrid type structure. This fact might be the key factor that contributes to the various topology changes. Interestingly, all three modifications originated a topology change for parallel type of the **ON2** variants when the modification was present within the loop, near guanosine residue.

### Antiproliferative properties of modified G-quadruplexes

Guanosine-rich sequences with the capacity to fold into G-quadruplex structures are well known for having superior antiproliferative potential in cancer cell lines [8, 11, 36, 37]. Even though their anticancer properties have been associated with targeting various proteins or toxicity of G-quadruplex degradation products, the detailed mechanism of action remains unclear. Chemical modifications, such as LNA or UNA, are able to modulate the biological properties of G-quadruplex structures [11, 19, 20]. In order to better understand the influence of the LNA, UNA and 2’-O-Me-RNA chemical modifications on the capacity of G-quadruplexes to inhibit cancer cells growth, the MTT assay in human cervical adenocarcinoma (HeLa) cell line was performed. This colorimetric technique allows to determine the cell viability through the reduction of the water-soluble yellow 3-[4,5-dimethylthiazole-2-yl]-2,5-diphenyltetrazolium bromide (MTT) to insoluble dark blue formazan. The amount of colorful product is directly proportional to the number of viable cells [38].

**Fig 6:**
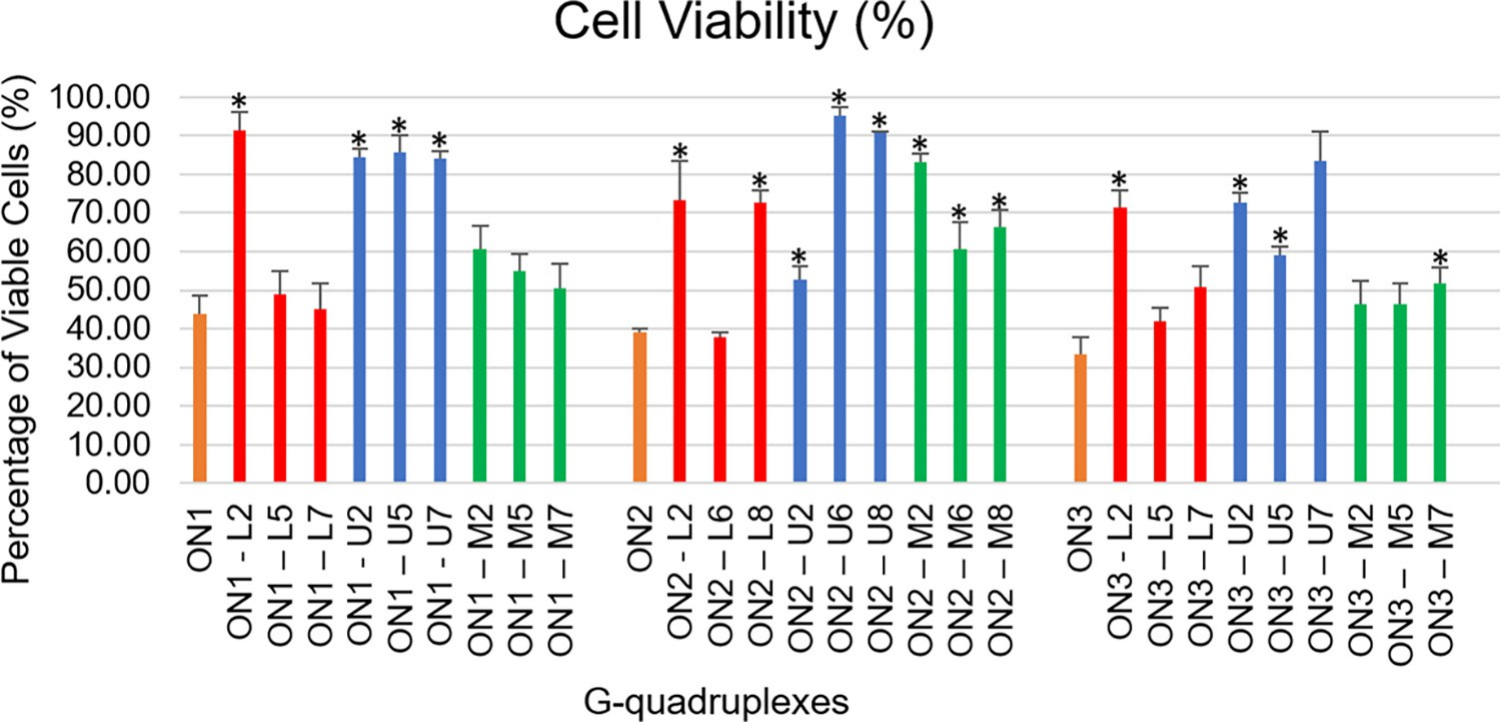
Antiproliferative activity of the G-quadruplex variants studied at 10 μM, using MTT assay. Orange–unmodified G-quadruplexes, red–LNA-modified G-quadruplexes, blue–UNA-modified G-quadruplexes, green– 2’-O-Me-RNA-modified G-quadruplexes. The statistic was performed based on comparison of the inhibitory effects after treatment with the unmodified sequence and treatment with modified variants. * *p* < 0.001 by one‐way ANOVA.

The antiproliferative activity of **ON1**, **ON2** and **ON3** G-quadruplexes was previously established as having significant inhibitory effect on HeLa cancer cell line growth, mounting 56%, 61% and 67%, respectively [5]. In general, none of the modified **ON1**, **ON2** and **ON3** variants showed improved antiproliferative activity (Fig 6). The majority of G-quadruplex variants modified with LNA residues within the loops show comparable inhibitory effect to parental, unmodified G-quadruplexes, with mostly no statistically significant difference when compared with the parental sequences. The **ON1–L5** and **ON1–L7** reduced HeLa cell viability up to 48.8% and 45.2%, respectively. Among LNA-modified **ON2** variants, only **ON2–L6** caused a decrease in HeLa cell viability up to 37.7%, whereas treatment with **ON2–L8** resulted in retaining 72.6% of viable *HeLa* cancer cells. The exposure of cells to **ON3–L5** and **ON7**–**L7** variants caused a decrease in HeLa cell growth up to 42.1% and 51.0%, respectively. In contrast, all three G-quadruplex variants modified with LNA residues within G-tetrads (**ON1**–**L2**, **ON2**–**L2**, **ON3**–**L2**) caused only minor, 10–30% inhibition of cancer cells growth. These LNA-modified G-quadruplexes were characterized by substantial improvement of the thermal stability, clearly demonstrating that the antiproliferative effect is not directly dependent on the thermal stability of the structure.

The treatment of cells with UNA-modified G-quadruplex variants resulted in increased *Hela* cells viability in comparison to the impact of parental sequences. In general, the presence of UNA within majority of studied G-quadruplexes caused a significant increase in the number of viable cells from 43.9, 39.0 and 33.3% for the unmodified **ON1**, **ON2** and **ON3** G-quadruplexes, respectively, to 70–90% for the variously modified variants. The only exception was **ON2–U2**, which inhibited cells growth by 47.3% (52.7% of viable cells). The above results clearly show that UNA modifications are unfavorable for the antiproliferative properties of the G-quadruplexes, being in accordance with the previous studies reported by Kotkowiak et al. indicating very moderate ability of UNAs to influence anticancer properties of TBA [11].

The presence of 2’-O-Me-RNA residue within G-tetrad of **ON1** (**ON1**–**M2**) resulted in a minor loss of **ON1** antiproliferative activity, decreasing HeLa cell viability up to 60.5%. In contrast, the presence of 2’-O-Me-RNA modification within **ON1** loops maintains the inhibitory potential of the G-quadruplex leading to cell viability at the level of 53.8% for **ON1–M5** and 50.6% for **ON1**–**M7**. For the **ON2** variants modified with 2’-O-Me-RNA we could observe that none of them maintain similar antiproliferative potential when compared with **ON2**. Treatment of cells with **ON3** variants modified with 2’-O-Me-RNA in the G-tetrad (**ON3–M2**) or in the middle of the loop (**ON3–M5**) maintains the growth inhibition capacity of **ON3** with no statistically significant difference when compared with the parental sequence. The presence of 2’-O-Me-RNA residue at position T7 of the loop (**ON3–M7**) demonstrated also certain antiproliferative potential, however the level of viable cells was slightly higher than for **ON3** (51.9% of viable cells).

The above data indicates that the LNA chemical modifications can be introduced in different loop positions without a significant influence on the antiproliferative potential of majority of the G-quadruplex variants. However, the presence of LNA residues is detrimental for the biological activity when modifications are placed in G-tetrads. All 2’-O-Me-RNA-modified variants of **ON1** and **ON3** demonstrated certain antiproliferative effect, whereas **ON2** variants showed substantially decreased inhibitory activity. Importantly, the presence of UNA residues within G-quadruplex structures appeared detrimental for inhibitory activity of the majority of **ON1–ON3** variants. The increased flexibility of G-quadruplex structures induced by the presence of UNA modifications might interfere with the favorable interactions between the G-quadruplexes and a target, being the reason for the decreased inhibitory effect observed for UNA-modified **ON1–ON3** variants.

### The influence of modified residues on enzymatic resistance of G-quadruplexes

The biological stability of G-quadruplexes is a key feature of therapeutic oligonucleotides. However, the degradation by exonucleases under physiological conditions is one of the main problems in applications of therapeutic oligonucleotides [7, 8, 11]. The introduction of chemical modifications, such as LNAs or UNAs can enhance the G-quadruplex physiological stability as well as the nuclease resistance [7, 11, 17]. The 2’-O-Me-RNAs can also significantly improve nuclease resistance of oligonucleotides [9].

**Fig 7:**
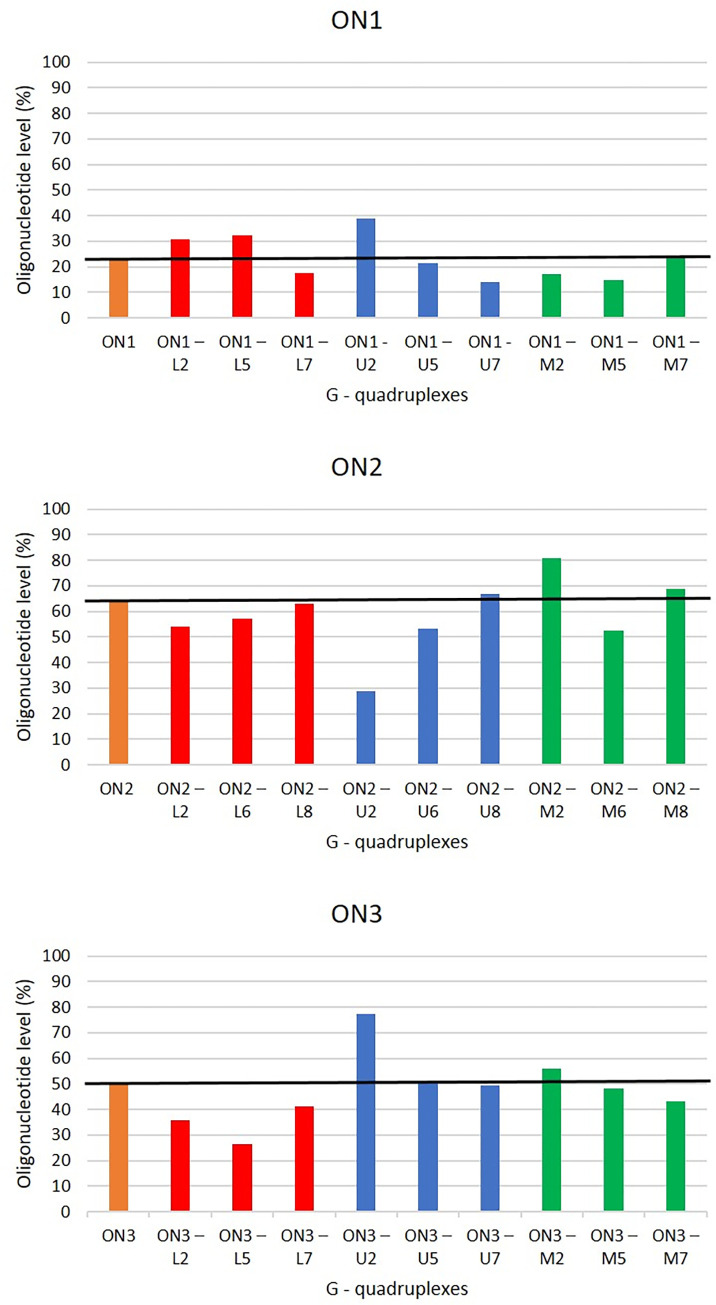
Level of undegraded G-quadruplex variants after 24h of incubation in RPMI medium supplemented with 10% of FBS at 37°C.

Nuclease stability assay can offer information about the inherent enzymatic stability of oligonucleotides providing valuable instructions for the G-quadruplex design [9, 11, 39]. The investigations of stability of all studied G-quadruplex variants were performed by CD spectroscopy in RPMI medium supplemented with 10% FBS and the degradation tendency was analyzed at time 0 and after 24h of incubation at 37°C. The level of undegraded parental oligonucleotides after 24h of exposure to RPMI medium was 22.7%, 64.6% and 50.5% for **ON1**, **ON2** and **ON3**, respectively (Table 2, Fig 7).

**Table 2:** Level of undegraded G-quadruplex variants*.

G-quadruplexes	Oligonucleotide level (%)	G-quadruplexes	Oligonucleotide level (%)	G-quadruplexes	Oligonucleotide level (%)
**ON1**	22.7	**ON2**	64.6	**ON3**	50.5
**ON1 –L2**	30.7	**ON2 –L2**	53.9	**ON3 –L2**	35.6
**ON1 –L5**	32.2	**ON2 –L6**	56.9	**ON3 –L5**	26.3
**ON1 –L7**	17.4	**ON2 –L8**	62.9	**ON3 –L7**	41.0
**ON1 –U2**	38.9	**ON2 –U2**	28.9	**ON3 –U2**	77.4
**ON1 –U5**	21.5	**ON2 –U6**	53.2	**ON3 –U5**	50.9
**ON1 –U7**	14.1	**ON2 –U8**	67.0	**ON3 –U7**	49.5
**ON1 –M2**	17.1	**ON2 –M2**	80.6	**ON3 –M2**	55.9
**ON1 –M5**	14.9	**ON2 –M6**	52.3	**ON3 –M5**	48.3
**ON1 –M7**	23.4	**ON2 –M8**	68.9	**ON3 –M7**	42.9

* After 24h of incubation in RPMI medium supplemented with 10% of FBS at 37°C.

The substitution of LNA modifications within **ON1** resulted in less than 10% increase in enzymatic stability for two of the G-quadruplex variants (**ON1–L2** and **ON1–L5**, Table 2, Fig 7). However, **ON1–L7** demonstrated 5.3% decrease in nuclease stability. The presence of LNA at T8 position of **ON2** (**ON2–L8**) did not change the oligonucleotide enzymatic resistance. However, a decrease of stability in the 8–11% range was observed for **ON2–L2** and **ON2–L6**. The presence of LNA within **ON3** decreased nuclease stability of parental G-quadruplex by 14.9% (**ON3–L2**), 24.1% (**ON3–L5**), and 9.5% (**ON3–L7**). In general, LNA modification did not demonstrated universal tendency for improvement of the nuclease stability of studied G-quadruplexes. Despite the above results, LNA rarely might be also a modification of choice for modulation of G-quadruplex properties, as presented for **ON1–L5** which was characterized by moderately enhanced enzymatic resistance without major influence on the antiproliferative potential of G-quadruplex.

The linear oligonucleotides are more prone to nuclease degradation than structuralized G-quadruplexes. Thus, susceptibility to degradation was expected for the G-quadruplex variants containing UNA-modified G-tetrads due to their low T_M_ [17]. Surprisingly, **ON1–U2** and **ON3–U2** demonstrated a significantly elevated levels of undegraded oligonucleotide after 24h in reference to unmodified parental compounds (Table 2, Fig 7). Nevertheless, UNA-modified loops within **ON1–U5** had no influence on nuclease stability, whereas 8.7% decrease in oligonucleotide level was observed for **ON1–U7**. The effect for UNA-modified loops within **ON2** variants was opposite. The **ON2–U6** possessed 11.4% decreased nuclease stability, whereas the stability of **ON2–U8** was slightly improved. UNA-modified **ON3** variants, **ON3–U5** and **U3–U7**, demonstrated similar nuclease stability to parental unmodified G-quadruplex. According to Agarwal et al. UNA modification provides exceptionally high serum stability when placed in the loop or even in the stem of G-quadruplex due to the lack of the C2’- C3’ bond within the ribose ring and thus becoming more resistant to the cleavage by nucleases [17]. Our studies do not fully support these findings, indicating that the effect is highly dependent on the G-quadruplex structure.

The 2’-O-Me-RNA-modified **ON1–M2** and **ON1–M5** demonstrated only minor decrease in undegraded oligonucleotide fraction (5.7% and 7.8%), whereas a similar stability tendency in comparison to unmodified **ON1** was observed for **ON1–M7**. The **ON2** variants modified with 2’-O-Me-RNA were characterized by 16.1% and 4.3% increase in the nuclease stability for **ON2**–**M2** and **ON2–M6**, respectively. In contrast, **ON2–M6** demonstrated a decrease of undegraded oligonucleotide level by 12.3%. The presence of 2’-O-Me-RNA residue within **ON3** G-tetrad (**ON3–M2**) induced minor, 5.4% increase of the G-quadruplex nuclease stability. However, the modification of the loops by 2’-O-Me-RNA led to a decrease in enzymatic stability by 2.2% and 7.5% for **ON3–M5** and **ON3–M7**, respectively.

The nuclease stability of the analyzed G-quadruplexes is neither directly proportional to their thermodynamic stability nor to their antiproliferative properties. The 2’-O-Me-RNA revealed to be the most neutral modification which enhances or maintains the G-quadruplex enzymatic resistance without significant depletion of the G-quadruplexes antiproliferative potential. Surprisingly, the presence of UNA within G-tetrads of two studied types of G-quadruplexes appeared favorable for the biological stability of these oligonucleotides despite a large destabilization of G-quadruplex structure and unfavorable changes of antiproliferative potential.

## Conclusions

Chemical modifications play important role in the G-quadruplexes design, since they can modulate their properties. In this study, the structural and biological properties of twenty-seven G-quadruplex variants containing LNA, UNA or 2’-O-Me-RNA modifications in the loop or in the G-tetrad were examined. As this was a preliminary evaluation of these novel G-quadruplexes, a single 10 μM concentration of oligonucleotides was used to provide some general conclusions to foster more detailed investigations. The above studies suggest that the G-quadruplex structural elements cannot be considered separately without consideration of their involvement in the interactions with other parts of the structure, therefore the structural prediction still represents a great challenge. The influence of each modification on the physicochemical properties of G-quadruplexes can be different, depending on mutual interactions between G-tetrads, loops, and in some cases also additional guanosine at 5’ or 3’ end. Moreover, the inhibitory activity of the G-quadruplexes is strongly dependent not only on the structure but also on local changes of residues chemical characteristics and on the specific interactions with the binding target. To summarize, UNA modifications can be modulators of G-quadruplex thermodynamic stability but are rather of little use for improvement of anticancer potential of studied G-quadruplexes. In contrast, 2’-O-Me-RNA modified G-quadruplexes were found to be effective in inhibition of HeLa cancer cells proliferation *in vitro*, without significant changes in the thermal stability, structure folding topology and with maintaining certain enzymatic resistance. Moreover, G-quadruplexes modified by LNAs within the loops demonstrated to have similar antiproliferative potential to the native sequences. Both modifications were confirmed to be modifications of choice for designing new potential G-quadruplex-based therapeutic candidates for future pre-clinical investigations.

## Supporting information

S1 File (DOCX)

## Abbreviations

2’-O-Me-RNA: 2’-O-methyl-RNA
MTT: 3-[4,5-dimethylthiazole-2-yl]-2,5-diphenyltetrazolium bromide
CD: circular dichroism
FBS: fetal bovine serum
G4s: G-quadruplexes
HeLa: human cervical adenocarcinoma
LNA: locked nucleic acid
KCl: potassium chloride
UNA: unlock nucleic acid
