# Supplementary material for: Changes in physicochemical and anticancer properties modulated by chemically modified sugar moieties within sequence-related G-quadruplex structures
Source: PLoS One. 2022 Aug 23;17(8):e0273528. doi: 10.1371/journal.pone.0273528 (PMC9397905; doi:10.1371/journal.pone.0273528)
Supplement: S1 File — (DOCX) [file pone.0273528.s001.docx]

| **Supporting Information**  **Evaluation of changes in physicochemical and anticancer properties modulated by chemically modified sugar moieties within sequence-related G-quadruplex structures**  Carolina Roxo and Anna Pasternak *  Department of Nucleic Acids Bioengineering, Institute of Bioorganic Chemistry, Polish Academy of Sciences, Noskowskiego 12/14, 61-704 Poznan, Poland  **To whom correspondence should be addressed. Tel: + 48 618 528 503; Email: apa@ibch.poznan.pl (A.P.);*   \| **Table S1.**  MALDI-MS data of oligonucleotides \| \| \| \| --- \| --- \| --- \| \| **Name** \| **Calculated oligonucleotide**  **mass** \| **MALDI-MS [M+H]^+^**  **m/z** \| \| ON1 \| 3130.1 \| 3131.95 \| \| ON1 - L2 \| 3157.1 \| 3158.05 \| \| ON1 – L5 \| 3157.1 \| 3158.12 \| \| ON1 – L7 \| 3157.1 \| 3158.69 \| \| ON1 - U2 \| 3132.1 \| 3146.24 \| \| ON1 – U5 \| 3132.1 \| 3132.89 \| \| ON1 - U7 \| 3132.1 \| 3133.66 \| \| ON1 – M2 \| 3144.1 \| 3159.33 \| \| ON1 – M5 \| 3144.1 \| 3145.45 \| \| ON1 – M7 \| 3144.1 \| 3145.72 \| \|  \|  \|  \| \| ON2 \| 3459.3 \| 3460.27 \| \| ON2 - L2 \| 3486.3 \| 3487.28 \| \| ON2 – L6 \| 3486.3 \| 3487.21 \| \| ON2 – L8 \| 3486.3 \| 3487.45 \| \| ON2 – U2 \| 3461.3 \| 3518.45 \| \| ON2 – U6 \| 3461.3 \| 3462.04 \| \| ON2 – U8 \| 3461.3 \| 3462.30 \| \| ON2 – M2 \| 3473.3 \| 3487.15 \| \| ON2 – M6 \| 3473.3 \| 3480.56 \| \| ON2 – M8 \| 3473.3 \| 3474.75 \| \|  \|  \|  \| \| ON3 \| 3459.3 \| 3460.20 \| \| ON3 - L2 \| 3486.3 \| 3487.62 \| \| ON3 – L5 \| 3486.3 \| 3488.55 \| \| ON3 – L7 \| 3486.3 \| 3487.48 \| \| ON3 – U2 \| 3461.3 \| 3477.34 \| \| ON3 – U5 \| 3461.3 \| 3462.24 \| \| ON3 – U7 \| 3461.3 \| 3462.30 \| \| ON3 – M2 \| 3473.3 \| 3490.04 \| \| ON3 – M5 \| 3473.3 \| 3474.25 \| \| ON3 – M7 \| 3473.3 \| 3474.74 \| | | | | | | | | | | | | |
| --- | --- | --- | --- | --- | --- | --- | --- | --- | --- | --- | --- | --- | --- | --- | --- | --- | --- | --- | --- | --- | --- | --- | --- | --- | --- | --- | --- | --- | --- | --- | --- | --- | --- | --- | --- | --- | --- | --- | --- | --- | --- | --- | --- | --- | --- | --- | --- | --- | --- | --- | --- | --- | --- | --- | --- | --- | --- | --- | --- | --- | --- | --- | --- | --- | --- | --- | --- | --- | --- | --- | --- | --- | --- | --- | --- | --- | --- | --- | --- | --- | --- | --- | --- | --- | --- | --- | --- | --- | --- | --- | --- | --- | --- | --- | --- | --- | --- | --- | --- | --- | --- | --- | --- | --- | --- | --- | --- | --- | --- | --- | --- | --- | --- | --- |
| **Table S2.** Thermodynamic parameters of G-quadruplex formation^a^. | | | | | | | | | | | | |
| Sequence (5'-3') | | Average of curve fits | | | |  | T_M_^-1^ vs log C_T_ plots | | | | | |
|  |  | **-ΔH˚ (kcal/mol)** | **-ΔS˚**  **(eu)** | **ΔG˚_37_ (kcal/mol)** | **T_M_ ^b^**  **(˚C)** |  | **-ΔH˚**  **(kcal/mol)** | **-ΔS˚**  **(eu)** | **-ΔG˚_37_ (kcal/mol)** | **T_M_^b^**  **(˚C)** | **ΔΔG˚_37_ (kcal/mol)** | **ΔT_M_^b^ (˚C)** |
| **ON1** | GGGTTTTGGG | 67.3 ± 4.5 | 193.2 ± 13.7 | -7.36 ± 0.21 | 45.0 |  | 57.6 ± 1.3 | 162.6 ± 3.9 | -7.13 ± 0.02 | 45.0 |  |  |
| **ON1-L2** | G **G^L^** GTTTTGGG | 83.6 ± 11.7 | 234.8 ± 33.3 | -10.78 ± 1.48 | 57.2 |  | 41.1 ± 0.8 | 105.8 ± 2.4 | -8.28 ± 0.04 | 58.0 | -1.15 | +13 |
| **ON1 – L5** | GGGT **T^L^** TTGGG | 63.5 ± 3.2 | 183.2 ± 9.1 | -6.70 ± 0.39 | 42.1 |  | 44.1 ± 2.6 | 121.5 ± 8.2 | -6.43 ± 0.05 | 42.4 | +0.7 | -2.6 |
| **ON1 – L7** | GGGTTT **T^L^** GGG | 72.7 ± 8.2 | 209.8 ± 23.1 | -7.60 ± 1.05 | 45.4 |  | 35.3 ± 0.8 | 91.9 ± 2.4 | -6.75 ± 0.02 | 46.7 | +0.38 | +1.7 |
| **ON1 - U2** | G **G^U^** GTTTTGGG | 39.8 ± 9.4 | 119.9 ± 32.7 | -2.66 ± 0.73 | 15.2 |  | 39.4 ± 5.634 | 118.1± 19.5 | -2.75 ± 0.45 | 15.6 | +4.38 | -29.4 |
| **ON1 – U5** | GGGT **U^U^** TTGGG | 70.5 ± 6.7 | 202.6 ± 19.9 | -7.68 ± 0.58 | 46.1 |  | 44.3 ± 1.6 | 120.6 ± 5.133 | -6.93 ± 0.04 | 46.1 | +0.27 | +1.1 |
| **ON1 – U7** | GGGTTT **U^U^**GGG | 76.1 ± 9.4 | 223.4 ± 27.7 | -6.85 ± 0.99 | 41.8 |  | 36.7 ± 1.0 | 98.2 ± 3.3 | -6.39 ± 0.01 | 43.1 | +0.74 | -1.9 |
| **ON1 – M2** | G **G^M^** GTTTTGGG | 71.9 ± 10.7 | 208.1 ± 31.2 | -7.35 ± 1.07 | 44.4 |  | 33.3 ± 0.9 | 86.4 ± 2.9 | -6.50 ± 0.02 | 44.9 | +0.63 | -0.1 |
| **ON1 – M5** | GGGT **U^M^** TTGGG | 68.7 ± 6.4 | 197.2 ± 19.7 | -7.55 ± 0.37 | 45.7 |  | 53.3 ± 0.7 | 148.8 ± 3.0 | -7.16 ± 0.02 | 45.9 | -0.03 | +0.9 |
| **ON1 – M7** | GGGTTT **U^M^** GGG | 68.4 ± 4.3 | 197.5 ± 12.9 | -7.13 ± 0.36 | 43.7 |  | 50.9 ± 1.1 | 142.5 ± 3.6 | -6.79 ± 0.02 | 43.9 | +0.34 | -1.1 |
| **ON2** | GGGGTTTTGGG | 72.4 ± 6.38 | 203.9 ± 16.6 | -9.15 ± 1.24 | 52.6 |  | 35.6 ± 3.7 | 90.9 ± 11.5 | -7.42 ± 0.20 | 53.0 |  |  |
| **ON2 – L2** | G **G^L^** GGTTTTGGG | 74.5 ± 8.3 | 207.1 ± 22.7 | -10.31 ± 1.29 | 57.6 |  | 37.5 ± 0.7 | 94.4 ± 2.2 | -8.17 ± 0.04 | 59.1 | -0.75 | +3.1 |
| **ON2 – L6** | GGGGT **T^L^** TTGGG | 62.0 ± 4.5 | 174.9 ± 13.4 | -7.78 ± 0.59 | 47.9 |  | 38.3 ± 1.4 | 100.5 ± 4.3 | -7.13 ± 0.04 | 49.3 | +0.29 | -3.7 |
| **ON2 – L8** | GGGGTTT **T ^L^** GGG | 77.9 ± 12.1 | 220.1 ± 34.9 | -9.69 ± 1.37 | 53.8 |  | 37.6 ± 1.4 | 96.5 ± 4.3 | -7.65 ± 0.06 | 54.2 | -0.22 | +1.2 |
| **ON2 – U2** | G **G^U^** GGTTTTGGG | 48.8 ± 2.2 | 140.1 ± 7.5 | -5.38 ± 0.17 | 35.1 |  | 42.0 ± 0.9 | 117.7 ± 2.9 | -5.51 ± 0.02 | 35.8 | +1.91 | -13.5 |
| **ON2 – U6** | GGGGT **U^U^** TTGGG | 73.8 ± 1.6 | 208.1 ± 3.8 | -9.27 ± 0.49 | 52.9 |  | 49.5 ± 2.3 | 133.1 ± 6.9 | -8.26 ± 0.09 | 54.0 | -0.82 | +1 |
| **ON2 – U8** | GGGGTTT **U^U^**GGG | 76.1 ± 8.8 | 216.3 ± 24.9 | -9.04 ± 1.14 | 51.3 |  | 37.5± 0.5 | 96.8 ± 1.4 | -7.43 ± 0.02 | 52.3 | -0.01 | -0.8 |
| **ON2 – M2** | G **G^M^** GGTTTTGGG | 68.3 ± 11.2 | 187.3 ± 32.4 | -10.25 ± 1.19 | 59.3 |  | 37.8 ± 2.9 | 95.5 ± 8.9 | -8.14 ± 0.21 | 58.6 | -0.72 | +5.6 |
| **ON2 – M5** | GGGGT **U^M^** TTGGG | 65.9 ± 6.1 | 186.1 ± 17.7 | -8.26 ± 0.69 | 49.6 |  | 41. ± 0.7 | 109. ± 2.3 | -7.41 ± 0.02 | 50.6 | +0.01 | -2.4 |
| **ON2 – M8** | GGGGTTT **U^M^** GGG | 67.3 ± 6.2 | 190.2 ± 17.1 | -8.34 ± 0.87 | 49.8 |  | 38.1 ± 0.9 | 99.3 ± 2.9 | -7.33 ± 0.03 | 51.1 | +0.09 | -1.9 |
| **ON3** | GGGTTTTGGGG | 64.9 ± 4.5 | 178.7 ± 14.0 | -9.51 ± 0.23 | 56.4 |  | 65.4 ± 5.5 | 180.0 ± 16.7 | -9.57 ± 0.32 | 56.6 |  |  |
| **ON3 –L2** | G **G^L^** GTTTTGGGG | 74.6 ± 8.0 | 205.7± 22.9 | -10.77 ± 0.96 | 59.7 |  | 47.1 ± 1.8 | 122.8 ± 5.6 | -9.00 ± 0.11 | 60.5 | +0.57 | +3.9 |
| **ON3 – L5** | GGGT **T^L^** TTGGGG | 74.2 ± 4.3 | 204.3 ± 12.8 | -10.8 ± 0.35 | 60.0 |  | 69.3 ± 2.1 | 189.6 ± 6.2 | -10.45± 0.13 | 60.0 | -0.88 | +3.4 |
| **ON3 – L7** | GGGTTT **T^L^** GGGG | 78.9 ± 3.4 | 222.6 ± 11.3 | -9.93 ± 0.20 | 54.7 |  | 60.1 ± 3.9 | 164.8± 12.0 | -9.01 ± 0.21 | 55.2 | +0.56 | -1.4 |
| **ON3 – U2** | G**G^U^**GTTTTGGGG | 41.5 ± 5.3 | 120.6 ± 16.9 | -4.11 ± 0.21 | 25.7 |  | 68.9 ± 18.6 | 211.9 ± 61.9 | -3.11 ± 0.88 | 25.8 | +6.46 | -30.8 |
| **ON3 – U5** | GGGT **U^U^** TTGGGG | 78.1 ± 4.4 | 216.1 ± 13.2 | -11.08 ± 0.35 | 60.1 |  | 67.4 ± 1.9 | 183.9 ± 5.7 | -10.40 ± 0.12 | 60.3 | -0.83 | +3.7 |
| **ON3 – U7** | GGGTTT **U^U^** GGGG | 72.4 ± 7.0 | 205.3 ± 20.5 | -8.73 ± 0.72 | 50.7 |  | 48.2 ± 2.0 | 130.3 ± 6.4 | -7.81 ± 0.07 | 51.3 | +1.76 | -5.3 |
| **ON3 –M2** | G **G^M^** GTTTTGGGG | 72.1 ± 3.8 | 201.8 ± 11.2 | -9.53 ± 0.32 | 54.5 |  | 59.5 ± 2.2 | 163.1 ± 6.8 | -8.90 ± 0.11 | 54.8 | +0.67 | -1.8 |
| **ON3 – M5** | GGGT **U^M^** TTGGGG | 74.0 ± 3.6 | 206.1 ± 10.6 | -10.10 ± 0.312 | 56.7 |  | 63.9 ± 1.8 | 175.2 ± 5.5 | -9.53 ± 0.09 | 56.9 | +0.04 | +0.03 |
| **ON3 – M7** | GGGTTT **U^M^** GGGG | 71.3 ± 3.5 | 198.5 ± 10.4 | -9.76 ± 0.28 | 55.8 |  | 63.6 ± 1.1 | 174.9 ± 3.4 | -9.33 ± 0.06 | 55.9 | +0.24 | -0.7 |
